# A Modified *o*-Phthalaldehyde Fluorometric Analytical Method for Ultratrace Ammonium in Natural Waters Using EDTA-NaOH as Buffer

**DOI:** 10.1155/2014/728068

**Published:** 2014-11-11

**Authors:** Hongzhi Hu, Ying Liang, Shuo Li, Qing Guo, Chancui Wu

**Affiliations:** ^1^School of Life and Environmental Sciences, Guilin University of Electronic Technology, Guilin 541004, China; ^2^School of Automation Engineering, University of Electronic Science and Technology of China, Chengdu 611731, China

## Abstract

In the existence of appropriate amount of disodium ethylenediaminetetraacetate (EDTA), precipitation would not occur in seawater and other natural waters even if the sample solution was adjusted to strong basicity, and the NH_3_-OPA-sulfite reaction at the optimal pH range could be used to determine ammonium in natural waters. Based on this, a modified *o*-phthalaldehyde fluorometric analytical method has been established to determine ultratrace ammonium in natural waters. Experimental parameters, including reagent concentration, pH, reaction time, and effect of EDTA, were optimized throughout the experiments based on univariate experimental design. The results showed that the optimal pH range was between 10.80 and 11.70. EDTA did not obviously affect the fluorometric intensity. The linearity range of the proposed method was 0.032–0.500 *µ*mol/L, 0.250–3.00 *µ*mol/L, and 1.00–20.0 *µ*mol/L at the excitation/emission slit of 3 nm/5 nm, 3 nm/3 nm, and 1.5 nm/1.5 nm, respectively. The method detection limit was 0.0099 *µ*mol/L. Compared to the classical OPA method, the proposed method had the advantage of being more sensitive and could quantify ultratrace ammonium without enrichment.

## 1. Introduction

Ammonia nitrogen consists of ammonia (NH_3_) and ammonium (NH_4_^+^) in natural waters. Ammonium is predominant when the pH is below 8.75, and ammonia is predominant when pH is above 9.75 [1]. Ammonium is the main species in the pH range of most natural waters and is an essential nutrient in aquatic ecosystems [2]. The concentration of ammonium is usually more than micromolar level in mostly continental water and coastal seawater, even up to millimolar level due to environmental pollution [3]. However, it is less than micromolar level, even down to nanomolar level in ocean water [4]. The accurate measurement of ammonium concentrations is fundamental to understanding nitrogen biogeochemistry in aquatic ecosystems. The most common techniques used to measure ammonium in freshwater and seawater are the indophenol blue method [5, 6] and* o*-phthalaldehyde (OPA) fluorometric method [7–15]. The* o*-phthalaldehyde (OPA) fluorometric method is much more sensitive than the indophenol blue method. It has attracted a great deal of attention of scientists. In 1971, it was firstly reported that OPA could react with amino acid and ammonium in the existence of mercaptoethanol to produce a strongly fluorescent compound [7]. In 1989, the reaction was modified by replacing mercaptoethanol with sulfite, organic amine compounds did not interfere in the determination, and an OPA fluorometric method with higher sensitivity and selectivity was developed for ammonium by Genfa and Dasgupta [8]. Afterwards, the method was further modified for the determination of ammonium in seawater [9–11] and was developed for shipboard using flow injection technology [12–14]. Recently, the sensitivity of the OPA method was further remarkably improved to determine ocean surface water by combining fluorescence detection with flow analysis and solid phase extraction [15]. The main analytical parameters of the OPA methods mentioned above were listed in Table 1. The lower limit of quantitation (LOQ) listed in the table was the lowest concentration of the working range in the corresponding references. Table 1 showed that the sensitivity of the reported methods was gradually improved and the LOQ was decreased using advanced technology such as flow injection, autonomous batch, and solid extraction technology.

The fluorometric reaction of NH_3_-OPA-sulfite was found to be pH dependent by much reported work. The optimal pH was reported at 11 by Amornthammarong and Zhang in 2008 [12]. However, when pH was more than 10.4, precipitation easily occurred due to the existence of metal ions in natural water sample. To avoid precipitation of the metal ions, much work had to control the pH about 9.3 using sodium tetraborate solution as buffer [9, 10, 14, 15]. According to the reported data, the sensitivity of the method at pH = 9.3 was several times less than that at pH = 11. Therefore, it was considered that the method sensitivity could be also proved by changing the reaction pH. In this work, a modified NH_3_-OPA-sulfite reaction using EDTA-NaOH as buffer was described. In the existence of appropriate amount of EDTA, precipitation would not occur in natural water even if the solution was adjusted to strong basicity, and the NH_3_-OPA-sulfite reaction under the optimal pH condition could be used to determine ammonium in natural waters. Based on this, a new modified* o*-phthalaldehyde fluorometric analytical method was established. The method is highly sensitive for determination of ammonium in natural waters without enrichment.

## 2. Experimental

### 2.1. Reagents and Solutions

All the chemicals used in this study were of analytical grade, supplied by Aladdin Chemical Reagent Co., China, unless stated otherwise. All solutions were prepared in ultrapure water (resistivity 18.2 MΩ·cm).

*Standard Solution.* Ammonium standard stock solution (1000 mg N/L) was purchased from Aladdin Chemical Reagent Co. Ammonium standard substock solution (10 mmol NH_4_^+^/L) was prepared monthly by diluting the stock solution with ultrapure water. The stock and substock solutions were stored at 4°C in a refrigerator while not in use. Ammonium working solution (0.1 mmol NH_4_^+^/L) was prepared daily by diluting 1.0 mL of the substock solution to 100 mL with ultrapure water.

*Fluorescent Reagent Solution (R*_*1*_ *).* 10.6 g/L OPA solution (Reagent A) was made by dissolving 2.65 g of OPA in 50 mL of methanol (HPLC grade) and diluted to 250 mL with ultrapure water. 2.5 g/L sodium sulfite solution (Reagent B) was made by dissolving 1.25 g of Na_2_SO_3_ in 500 mL ultrapure water and adding 0.20 mL HCHO to prevent the solution from being oxidized. To reduce reagent blank, fluorescent reagent solution (R_1_) was prepared daily by mixing equal volumes of Reagent A with Reagent B and allowed to stand in room temperature for at least 2 hours and then passed through the OASIS HLB 6 cc/200 mg cartridge (Waters Corp., Millford, MA) at a flow rate of 3.0 mL/min before use.

*EDTA-NaOH Buffer Solution (R*_*2*_ *) and NaOH Solution.* EDTA-NaOH buffer solution (R_2_) was made by dissolving 46.6 g disodium ethylenediaminetetraacetate (EDTA, ACS grade) and 8.75 g NaOH (ACS grade) in 500 mL ultrapure water. NaOH solution (R_3_) was made by dissolving 10.0 g NaOH (ACS grade) in 500 mL ultrapure water. Sodium tetraborate buffer solution (R_4_) was prepared by dissolving 3.75 g Na_2_B_4_O_7_·10H_2_O in 500 mL ultrapure water. These above-mentioned three solutions were separately boiled for several ten minutes to remove ammonia till the final volume of the solution was reduced to half of the original volume and were immediately cooled in water bath and tightly sealed in a polypropylene bottle. The bottles were double-bagged using polyethylene bag while not in use.

All vessels used in the experiments were firstly soaked with 1 mol·L^−1^  HCl for more than 12 hours and cleaned with RO water and then were soaked with 1 mol·L^−1^  NaOH at least for 12 hours and cleaned thoroughly with ultrapure water before use.

### 2.2. Analytical Procedures

#### 2.2.1. The Proposed Method

20 mL of standard ammonium solution or sample solution with a concentration range of 0.032–15.0 *μ*mol/L was exactly measured into a polypropylene bottle. Appropriate amounts of fluorescent reagent (R_1_) and buffer (R_2_) were added into the solution. The concentrations of OPA, sodium sulfite, and EDTA in the final solution were 0.691 g/L, 0.163 g/L, and 5.77 mmol/L, respectively. The pH of the final solution was controlled in the range of 11.0–11.4 by adding appropriate amounts of NaOH solution (R_3_). After all the reagents were added, the mixed solution was tightly sealed and allowed to react for 50 minutes at room temperature. At least ten samples could be determined at the same time. The fluorescence intensity (FI) was measured on a fluorescence spectrophotometer (RF-5301PC, SHIMADZU Co., Ltd., Japan) with excitation wavelength set at 361 nm and emission wavelength at 423 nm.

#### 2.2.2. The Classical OPA Method

Based on the method of [10, 15], 20 mL of standard ammonium solution or sample solution with a concentration range of 0.250–2.00 *μ*mol/L was exactly measured into a polypropylene bottle. Both 3 mL of fluorescent reagent (R_1_) and 5 mL of sodium tetraborate buffer (R_4_) were added to the solution. After all the reagents were added, the mixed solution was tightly sealed and allowed to react for 60 minutes at room temperature. The pH of the reaction solution is about 9.3. The fluorescence intensity (FI) was measured in the same instrument as the proposed method.

## 3. Results and Discussion

### 3.1. Parameters Optimization

The OPA-NH_3_-sulfite reaction may be affected by the parameters OPA and sodium sulfite concentrations, reaction time, and pH. These parameters had been optimized by much work [7–10]. However, the reaction system in this work was different from previous works due to adding EDTA-NaOH buffer solution, so they were optimized based on a univariate experimental design again.

#### 3.1.1. Spectral Characteristics of the Reaction Production

**Figure 1:**
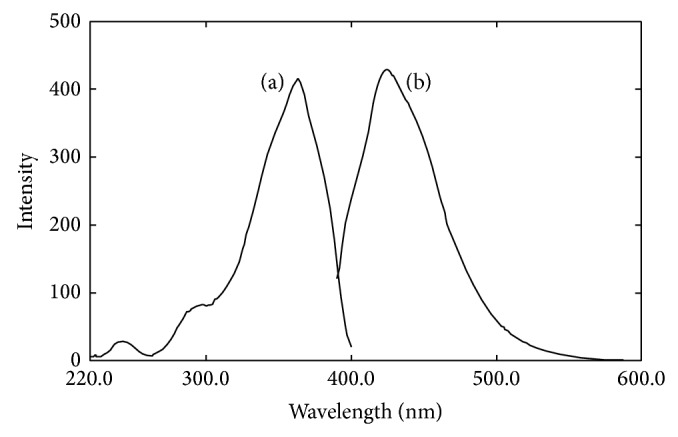
Excitation (a) and emission (b) spectra of products of OPA-NH_3_-sulfite (pH 11.3) in the presence of EDTA.

0.25 *μ*mol/L standard ammonium solution was allowed to react with OPA and sodium sulfite in the presence of EDTA-NaOH buffer according to Section 2.2.1. The excitation and emission spectra of the reaction product are showed in Figure 1.

The product had the maximum excitation wavelength (*λ*_ex_) at 361 nm and the maximum emission wavelength (*λ*_em_) at 423 nm, showing similar spectral characteristics as reported by Genfa and Dasgupta (1989). The results indicated that the spectral characteristics were not affected by EDTA. The time necessary for completion of the OPA-sulfite-NH_3_ reaction under this condition was about 50 min at room temperature. The excitation wavelength, emission wavelength, and reaction time were chosen as 361 nm, 423 nm, and 50 min in the following investigations, respectively.

#### 3.1.2. Effect of pH on the Reaction of OPA-NH_3_-Sulfite

**Figure 2:**
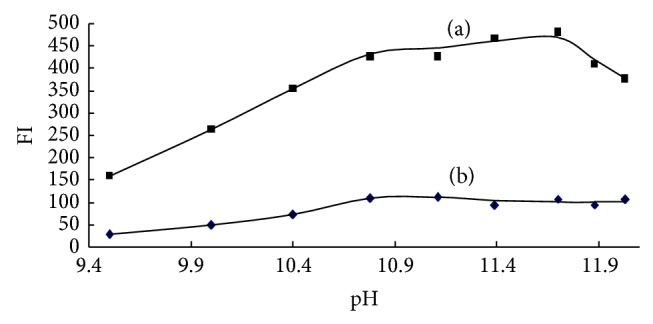
Effect of pH on the reaction of OPA-NH_3_-sulfite ((a) 0.25 *μ*mol/L, (b) blank).

It was reported that the OPA-NH_3_-sulfite reaction could be pH dependent and formed a fluorescent isoindole complex [16]. The optimal pH was at the range of 10–10.5 reported by Kuo et al. [17] and at 11 reported by Amornthammarong and Zhang [12]. The optimal range of pH reported in different paper was not completely uniform, but it was agreed that the pH had obvious influence on the formation of fluorescent isoindole complex. In this work, the effect of pH that varied from 9.5 to 12.0 was investigated. As showed in Figure 2, the fluorescence intensity (FI) increased with increasing pH from 9.5 up to 10.80, the maximum FI was observed in the pH range of 10.80–11.70, and then the FI decreased when the pH was higher than 11.70. The maximum FI was about three times of that of pH = 9.5. The variation trend and optimal pH range are similar to those of Amornthammarong and Zhang [12]. To gain better method sensitivity, the solution pH was controlled in the range of 11.0–11.4 using EDTA-NaOH buffer solution in this work. The EDTA effect was discussed in detail in Section 3.1.4.

#### 3.1.3. Effect of Concentrations of OPA and Sodium Sulfite

**Figure 3:**
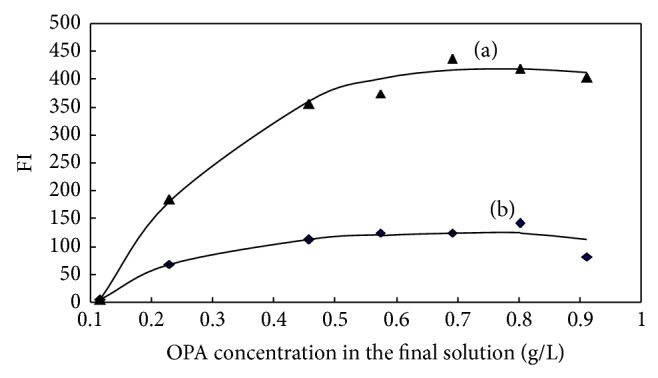
Effect of OPA concentration in the final solution on the reaction of OPA-NH_3_-sulfite ((a) 0.25 *μ*mol/L, (b) blank).

The effect of OPA concentration on the fluorescence reaction was studied over the range 0.115–0.913 g/L (Figure 3). The fluorescence intensity increased with increasing OPA concentration from 0.115 up to 0.457 g/L and was kept stable when the OPA concentration was between 0.457 and 0.913 g/L. Consequently, an OPA concentration of 0.691 g/L in the final solution was chosen for all subsequent experiments.

**Figure 4:**
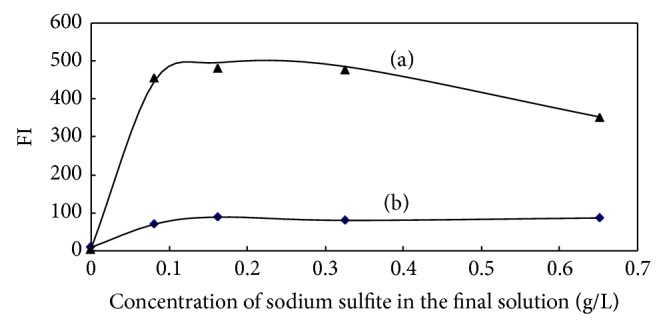
Effect of sodium sulfite concentration in the final solution on the reaction of OPA-NH_3_-sulfite ((a) 0.25 *μ*mol/L, (b) blank).

The effect of sodium sulfite in the range of 0–10 g/L is illuminated in Figure 4. The fluorescence intensity increased rapidly when concentration of sodium sulfite increased from 0 to 0.081 g/L and was kept constant in the range of 0.081–0.326 g/L, and then the intensity slowly decreased when the sodium sulfite concentration was more than 0.326 g/L. Therefore, a sodium sulfite concentration of 0.163 g/L in the final solution was selected in this work.

In this work, OPA and sodium sulfite were found to be the main source of reagent blank. To decrease the reagent blank, OPA and sodium sulfite solution were mixed together according to the depiction in Section 2.1 and passed through a HLB cartridge. In the mixed solution, ammonium in the reagent reacted with excess OPA and sodium sulfite and produced isoindole complex. The isoindole complex could be extracted by HLB column, ammonium in the solution was removed. Most of OPA and sodium sulfite in the solution passed through the column. The passed solution was used as fluorescent reagent. It was stable for one week if stored at 4°C in a refrigerator, while the reagent blank will slowly increase because of the effect of ambient air. To get lower reagent blank, the mixed solution was made daily in this work.

#### 3.1.4. Effect of EDTA

EDTA is a strong metal ion complexing agent. Precipitation would not occur in seawater sample at the existence of appropriate number of EDTA even if the solution was adjusted to strong basicity. When 3 mL of EDTA-NaOH buffer solution (R_2_) was added in 20 mL seawater sample in this work, the pH of the solution could be adjusted in the range of 11.0–11.4 and precipitation could not appear. The concentration of EDTA in the final solution was 5.77 mmol/L. This dosage was also suitable for determining freshwater sample. To investigate the effect of EDTA, the FI of different concentration standard ammonium solution was separately determined in the existence or absence of EDTA. The results are listed in Table 2. There was not an obvious difference between the FI signals of existence and absence of EDTA, illuminating that EDTA had not obviously influence the fluorimetric determination of ammonium. EDTA could be used to prevent metal ion precipitation in this proposed method.

### 3.2. Calibration Curves, Sensitivity, Reproducibility, and Method Detection Limit

Under the optimal conditions chosen above, the typical calibration curves were determined according to Section 2.2.1. The results are listed in Table 3. The linearity range was 0.032–0.500, 0.250–3.00, and 1.00–20.0 *μ*mol/L at the excitation/emission slit of 3 nm/5 nm, 3 nm/3 nm, and 1.5 nm/1.5 nm, respectively. The calibration curve with different linearity range could be used for typical sample analysis, depending on the concentration of ammonium in water samples.

Under the same experimental environment as the proposed method, a calibration curve of the classical OPA method was determined according to Section 2.2.2 and was FI = 312.6*C*_*N*_ + 60.7 at the excitation/emission slit of 3 nm/5 nm (Table 3). The slope of the proposed method is 1237.6, being 4 times that of the classical OPA method. This illuminated that the proposed method was much more sensitive than the classical method.

The reproducibility of the method was evaluated with 5 repetitive determinations of a 0.250 *μ*mol/L ammonium standard solution. The relative standard deviation was 3.2%. Eleven blanks solutions were determined at the excitation/emission slit of 3 nm/5 nm, the average FI was 45.71, and the standard deviation was 4.09. The method detection limit, estimated as three times the standard deviations of the blank, was 0.0099 *μ*mol/L.

### 3.3. Validation of the Method

#### 3.3.1. Recovery

Fresh water samples, groundwater and mountain spring water, were collected at Yaoshan Scenic Area in Guilin. A surface seawater sample was collected from the South China Sea and aged for one year. In order to examine the recovery of the method, these three samples spiked with a series of concentration of ammonium (0, 0.125, 0.250, 0.500, and 1.000 *μ*mol/L) were separately analyzed using the proposed method, together with the calibration curve. The linear equations of the matrix spike curves and corresponding calibration curves are showed in Table 4. The slopes of the calibration curves were not completely identical due to the slight difference of the room temperature and other environmental parameters in different days. To avoid the influence of the experimental environment, the matrix spiked curve and the corresponding calibration curve were determined in the same time. The recovery of the ammonium in spiked samples was represented as the ratio of the slope of matrix spike curve to that of corresponding calibration curve [18]. The average recovery of the ammonium in fresh water and seawater matrix ranged from 97.60% to 103.83%, illuminating that the other amine in the water samples did not disturb the determination of ammonium. The proposed method is available for both fresh water and seawater.

#### 3.3.2. Comparison with Classical OPA Method

Two typical seawater samples obtained from the South China Sea were analyzed using the proposed method according to Section 2.2.1. At the same time, the ammonium concentrations were determined using classical OPA method according to Section 2.2.2. The results are compared in Table 5. Using the paired Student's* t*-test at 95% confidence level to test the difference between the two methods, the calculated* t*-values were lower than the critical* t*-value. This indicates that there was no statistically significant difference between the proposed method and classical OPA method.

#### 3.3.3. Application

**Figure 5:**
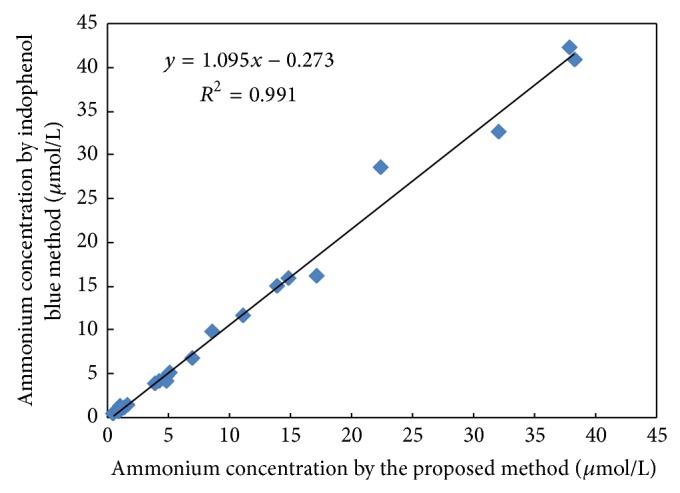
Intercomparison data with indophenol blue method.

**Figure 6:**
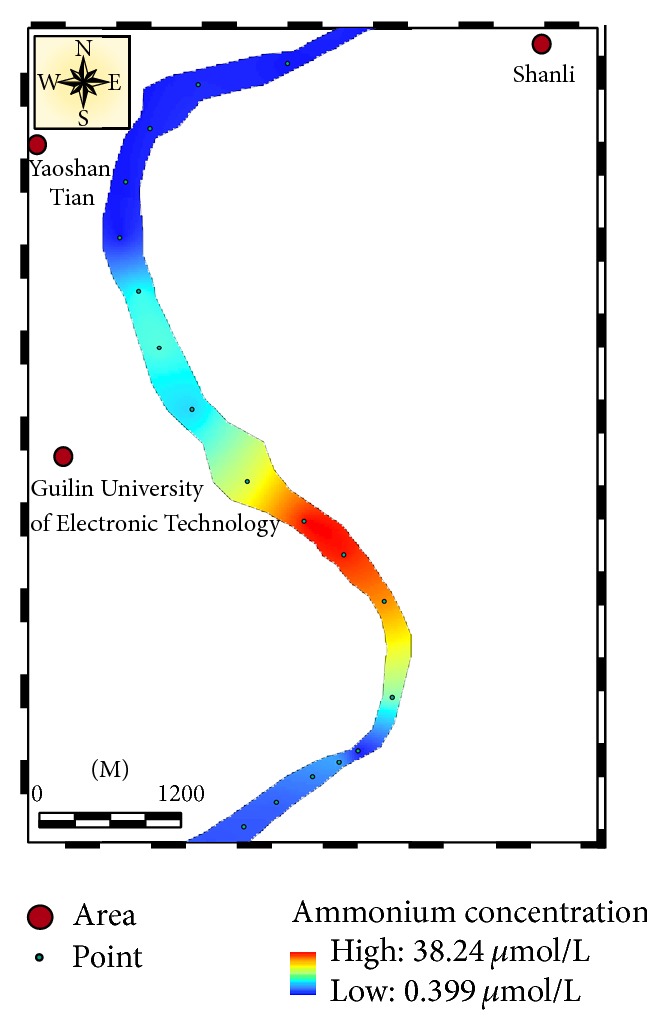
The spatial variation of ammonium concentration in Huajian River.

Huajian River is located in Guilin city and passes through Huajian Compus of Guilin University of Electronic Technology (GUET). Twenty-three surface water samples were collected from Huajian River at December 27, 2013, and filtered by 0.45 *μ*m filter as soon as possible after collection. The filtered water samples were refrigerated at 4°C before determination. The samples were analyzed by both the proposed method and indophenol blue method [5] within 24 hours. When the concentration of ammonium in the water sample was less than 15 *μ*mol/L, the FI of the water sample was determined according to Section 2.2.1, and the calibration curve with appropriate linearity range in Table 3 was applied to quantify the ammonium concentration. Otherwise, the water sample should be diluted before determination. The results in Figure 5 show a good agreement between these two methods with a wide concentration range from 0.44 to 38.25 *μ*mol/L. The spatial variation of ammonium in Huajian River is described in Figure 6. The higher concentrations of ammonium were found in the River at about 500 m downstream of GUET. The concentration of ammonium in the upstream was lower and decreased gradually as the distance to the GUET was increasing. This illuminated that the outfall of GUET was the most possible main source of ammonium in the river.

## 4. Conclusion

A new modified OPA fluorometric analytical method was established to determine ultratrace concentrations ammonium in natural waters using EDTA-NaOH as buffer. In this method, the NH_3_-OPA-sulfite reaction at the optimal pH could be used to determine ammonium in natural waters. There was no significant statistical difference between the results obtained from the proposed method and classical OPA method. The results of the proposed method applied to determine the river water were agreed with that of indophenol blue method. Compared to the classical OPA method, the main merit of the proposed method was enhancing the sensitivity by increasing the amount of reaction production under the optimal pH condition. It could quantify nanomolar level ammonium without enrichment.

## Figures and Tables

**Table 1:** The main analytical parameters of the typical reported OPA methods.

Samples	Technology	Reagent	Reaction temperature	Working range (nmol/L)	LOQ (nmol/L)	Reference
Standard solution	Manual	OPA, mercaptoethanol	Room temperature	1.0 × 10^5^–1.0 × 10^6^	1.0 × 10^5^	[7]
Fresh water	Flow injection	OPA, Na_2_SO_3_, phosphate	85°C	250–20000	250	[8]
Fresh/saline waters	Flow injection	OPA, Na_2_SO_3_, tetraborate	30°C	250–50000	250	[9]
Seawater	Manual	OPA, Na_2_SO_3_, tetraborate	Room temperature	ND–10000	ND	[10]
Seawater	Gas diffusion	OPA, Na_2_SO_3_	70°C	ND–40000	ND	[11]
Seawater	Flow injection	OPA, Na_2_SO_3_	65°C	100–600	100	[12]
Seawater	Autonomous batch analyzer	OPA, Na_2_SO_3_	Room temperature	200–1000	200	[13]
Seawater	Multipumping analyzer	OPA, Na_2_SO_3_, tetraborate	63.5–86.5°C	13–1000	13	[14]
Seawater	Solid extraction technology	OPA, Na_2_SO_3_, tetraborate	75°C	1.67–300	1.67	[15]

^*^LOQ: the lower limit of quantitation, the lowest concentration of the standard curves or working range reported in the corresponding reference.

ND means “no data.”

**Table 2:** The FI of different concentration standard ammonium solution in existence and absence of EDTA.

Concentration of ammonium (*C*_*N*_, *μ*mol/L)	FI in existence of EDTA	FI in absence of EDTA

0	62.871	74.994
0.125	253.749	235.408
0.250	444.613	414.782
0.375	589.166	551.247
0.500	760.241	690.585

The relationship between FI and *C*_*N*_	FI = 1384.1*C*_*N*_ + 76.10(*n* = 5)	FI = 1237.6*C*_*N*_ + 84.00(*n* = 5)

Corresponding parameters		
*R* ^2^	0.9973 (*P*< 0.0001)	0.9970 (*P*< 0.0001)
The standard deviation of the intercept	12.65	11.90
The standard deviation of the slope	41.32	38.87

**Table 3:** Calibration curves and the corresponding performances.

Method	Excitation/emission slit widths	Calibration curves	*n*	*R* ^2^	Standard deviation of the intercept	Standard deviation of the slope	Linearity range (*μ*mol/L)
	3 nm/5 nm	FI = 1237.6*C*_*N*_ + 84.0	6	0.9970	11.90	38.87	0.032–0.500
The proposed method	3 nm/3 nm	FI = 283.0*C*_*N*_ + 35.8	6	0.9996	4.88	2.94	0.25–3.00
	1.5 nm/1.5 nm	FI = 36.6*C*_*N*_ + 20.7	8	0.9971	5.58	0.81	1.00–15.0

The classical method	3 nm/5 nm	FI = 312.6*C*_*N*_ + 60.7	6	0.9951	5.71	12.66	0.25–2.00

**Table 4:** The matrix spiked recovery.

Matrix	Matrix spiked curve	Corresponding calibration curve	The average matrix spiked recovery

Groundwater	FI = 1240*C*_*N*_ + 162.0 (*n* = 5, *R* ^2^ = 0.9961)	FI = 1220*C*_*N*_ + 125.2 (*n* = 5, *R* ^2^ = 0.9955)	101.60%

Mountain spring water	FI = 1194*C*_*N*_ + 246.2 (*n* = 5, *R* ^2^ = 0.9908)	FI = 1150*C*_*N*_ + 172.0 (*n* = 5, *R* ^2^ = 0.9941)	103.83%

Seawater	FI = 915.1*C*_*N*_ + 118.2 (*n* = 5, *R* ^2^ = 0.9912)	FI = 937.8*C*_*N*_ + 119.4 (*n* = 5, *R* ^2^ = 0.9923)	97.60%

**Table 5:** Analytical results of the proposed method and classical OPA method.

Seawater sample	The proposed method (*μ*mol/L)	The classical OPA method (*μ*mol/L)	Calculated *t*-value	Critical *t*-value (*P* = 0.05)
1	0.536 ± 0.008 (*n* = 4)	0.560 ± 0.018 (*n* = 4)	2.43	2.45
2	0.385 ± 0.006 (*n* = 3)	0.360 ± 0.035 (*n* = 3)	1.22	2.78
